# Long noncoding RNA WT1-AS regulates trophoblast proliferation, migration, and invasion via the microRNA-186-5p/CADM2 axis

**DOI:** 10.1515/med-2022-0595

**Published:** 2022-12-06

**Authors:** Qun Qiu, Juan Tan

**Affiliations:** Department of Medical Genetics and Prenatal Diagnosis, Lianyungang Maternity and Child Health Hospital, Lianyungang 222000, China; Lianyungang Maternity and Child Health Hospital, No. 669 Qindongmen Street, Haizhou District, Lianyungang 222000, China; Maternal and Child Health Teaching and Research Section, Lianyungang Branch of Traditional Chinese Medicine, Jiangsu Union Technical Institute, Lianyungang 222000, China

**Keywords:** preeclampsia, placental trophoblasts, WT1 antisense RNA, microRNA-186-5p/cell adhesion molecule 2 axis

## Abstract

This study aimed to determine the role of long noncoding RNA (lncRNA) WT1 antisense RNA (WT1-AS) in the occurrence and progression of preeclampsia (PE) and to determine the underlying molecular mechanisms. The associations between WT1-AS and microRNA (miR)-186-5p, and miR-186-5p and cell adhesion molecule 2 (CADM2) were predicted using StarBase software and verified via dual-luciferase assays. To explore the role of the human chorionic trophoblast line HTR-8/SVneo, gene (WT1-AS/miR-186-5p) gain/loss of function experiments were performed. Qualitative reverse transcription-polymerase chain reaction (RT-PCR) analysis was used to evaluate transfection efficiency. Cell proliferation, apoptosis, cell migration, and invasion were assessed using 3-(4,5-dimethyl-2-thiazolyl)-2,5-diphenyl-2-*H*-tetrazolium bromide (MTT), flow cytometry, and transwell analysis, respectively. Moreover, CADM2 protein expression was measured by western blotting. The results indicated that overexpression of WT1-AS inhibited cell viability, migration, and invasion, and induced apoptosis in HTR-8/SVneo cells. We observed that miR-186a-5p directly targeted WT1-AS, and miR-186a-5p knockdown reversed the effects of WT1-AS knockdown in HTR-8/SVneo cells. Binding sites were found between miR-186-5p and CADM2, and CADM2-overexpression reversed the influence of miR-186-5p mimic on HTR-8/SVneo cells. In summary, our findings demonstrated that lncRNA WT1-AS participates in PE by regulating the proliferation and invasion of placental trophoblasts, through the miR-186-5p/CADM2 axis.

## 1 Introduction

Preeclampsia (PE), a disease that affects approximately 5% of all pregnancies, is one of the main causes of maternal morbidity worldwide [1,2]. PE is divided into mild and severe forms. In mild PE, pregnant women have high blood pressure and persistently increased proteinuria [3]. In addition to high blood pressure, severe PE symptoms include damage to other organs, including eclampsia, HELLP (hemolysis, elevated liver enzymes, thrombocytopenia) syndrome, liver damage, heart failure, abnormal kidney function, and fetal growth restriction [4,5,6]. A previous study demonstrated that abnormal placental development in early pregnancy could be a vital factor in the development of PE [7]. However, the pathogenesis of PE remains unclear.

Abnormal placental function, impaired trophoblast invasion, abnormal spiral artery remodeling, endothelial dysfunction, and promoted trophoblast apoptosis are associated with PE pathogenesis [8]. Among these, trophoblast behavior disorder is considered vital in the development of PE; thus, understanding its molecular mechanism can help develop novel treatment methods for PE [9]. Previous reports have demonstrated that the occurrence of severe PE is closely associated with the decline of trophoblast invasion and the failure of uterine spiral arteriole remodeling [10,11]. Failed spiral artery remodeling leads to reduced or abnormal uteroplacental perfusion, hypoxia, and damage to the syncytiotrophoblast, causing the release of factors such as SFlt-1, which induce endothelial cell dysfunction [12]. Moreover, uterine natural killer cells and macrophages are also involved in spiral artery remodeling, as are the extravillous trophoblasts (EVTs) [13]. It should be noted that EVTs fail to invade and remodel the spiral arteries in the first trimester of pregnancy [14]. In this study, therefore, we used placental trophoblasts to investigate PE.

Long noncoding RNAs (lncRNAs) are a class of single RNA molecules more than 200 nt in length, which do not encode for proteins but are involved in several regulatory processes, such as epigenetic regulation, transcription regulation, and posttranscriptional regulation [15,16]. MicroRNAs (miRNAs/miRs) are small, single-stranded RNA molecules with a length of 21–23 nt and are involved in cell differentiation, embryonic development, and disease occurrence and development [17–19]. lncRNAs can act as miRNA sponges, repressing miRNA expression and regulating mRNA expression at the posttranscriptional level. The analysis of mechanisms of miRNAs and lncRNAs action, and the use of the latest technology to investigate the association between miRNAs, lncRNAs, and diseases, has suggested that miRNAs and lncRNAs may be useful as novel biological markers for disease diagnosis and could provide new avenues for the treatment of diseases. Reports by Lv et al. suggested that the abnormal expression of placental lncRNAs and miRNAs may be associated with the occurrence and progression of PE [20–22]. In addition, increasing evidence suggests that miRNAs influence the behavior of placental trophoblasts by regulating the expression of their target genes, and participate in the progression of PE, playing an important role in its pathogenesis [23,24].

Previous studies have revealed that the migration capability of EVTs is regulated by several lncRNAs, such as maternally expressed 3 (lncRNA MEG3), colorectal neoplasia differentially expressed (lncRNA CRNDE), and growth arrest specific 5 (lncRNA-GAS5) [25–27]. The lncRNA WT1 antisense RNA (WT1-AS), an antisense transcript of Wilms tumor genes, regulates the invasiveness of multiple cells [28] and has been shown to be a vital regulator of cell proliferation, invasion, and migration [29–31]. Cui et al. suggested that WT1-AS suppressed cervical carcinoma cell proliferation, migration, and invasion through regulating the miR-330-5p/p53 axis [32]. However, the role of WT1-AS in the functioning of placental EVTs remains unknown. Wang et al. have reported that miR-186-5p is involved in various types of diseases, including ischemic stroke, atherosclerosis, diabetic cardiomyopathy, and cancer [33–36]. MiR-186-5p plays a role in disease development by regulating cell growth, invasion, migration, and apoptosis [33–36]. Previous studies have suggested that miR-186-5p is significantly increased in blood plasma during early-onset PE, and it plays a key role in the regulation of trophoblast cell viability [37,38]. The specific functions of miR-186-5p in PE and trophoblasts remain to be studied. Cell adhesion molecule 2 (CADM2), a member of the CADM family, has been found to maintain cell polarity, and previous studies have demonstrated that CADM2 could promote the migration and invasion of cancer cells, including those of endometrial cancer [39,40]. The role of CADM2 in EVT function remains to be explored. Thus, the miR-186-5p/CADM2 axis may interfere with PE through the regulation of the function of placental EVTs.

We hypothesized that lncRNA WT1-AS might affect trophoblast’s function in PE via regulating miR-186-5p/CADM2 axis. Hence, the purpose of this study was to investigate the effects of lncRNA WT1-AS on the function of HTR-8/Svneo cells and explore its possible involvement in the progression of PE, as well as to discover avenues for developing novel treatments for this condition.

## 2 Materials and methods

### 2.1 Cell culture

The human chorionic trophoblast line, HTR-8/Svneo, was obtained from American Type Culture Collection (ATCC, USA) and cultured in high-glucose Dulbecco’s modified Eagle medium (DMEM) supplemented with 10% fetal bovine serum (FBS; Gibco, USA) with 5% CO_2_ at 37°C. Graham et al. developed the HTR-8/SVneo cell line (https://web.expasy.org/cellosaurus/CVCL_7162) [41]. A recent study showed that the cell line consists of two populations: trophoblast and stromal/mesenchymal cells [42]. 293T cells were obtained from ATCC (MA, USA) and cultured in DMEM supplemented with 10% FBS (Gibco, USA) with 5% CO_2_ at 37°C.

### 2.2 Dual-luciferase reporter assay [43]

The StarBase software (version 2.0; https://starbase.sysu.edu.cn/) was used to investigate the association between WT1-AS and miR-186a-5p, or miR-186a-5p and CADM2. To confirm the association between WT1-AS and miR-186a-5p, the 3′-untranslated region (UTR) of WT1-AS was obtained via PCR, including its target sequence. The 3′-UTR was fused with the pmirGLO vector (Promega, USA) to construct the WT1-AS wild-type (WT1-AS-WT) reporter vector and the WT1-AS mutant (WT1-AS-MUT) vector. A total of 293T cells (5 × 10^4^ cells per well; American Type Culture Collection, USA) cultured for 24 h were co-transfected with WT1-AS-WT or WT1-AS-MUT luciferase reporter gene plasmids and miR-186a-5p mimic or mimic control for 48 h, using Lipofectamine^®^ 2000 reagent (Invitrogen, USA), in accordance with the manufacturer’s protocol. After 24 h, the Dual-Luciferase Reporter Assay System (Promega, USA) was used to assess the luciferase activity.

### 2.3 Cell transfection

HTR-8/Svneo cells (5 × 10^4^ cells per well) were cultured in six-well plates overnight and subsequently transfected with control plasmid, control-small interfering (si) RNA (Guangzhou Ribobio Co., Ltd., China), 100 nM inhibitor control (5′-GCCUCCGGCUUCGCACCUCU-3′; Shanghai GenePharma Co., Ltd., China), 100 nM mimic control (5′-UUCUCCGAACGUGUCACGUTT-3′; Shanghai GenePharma Co., Ltd.), WT1-AS plasmid, WT1-AS-siRNA (cat no. siG180524011008-1-5; Guangzhou Ribobio Co., Ltd.; https://www.ribobio.com/product_detail/?sku=siG180524011008-1-5), 100 nM miR-186-5p inhibitor (5′-AGCCCAAAAGGAGAAUUCUUUG-3′; Shanghai GenePharma Co., Ltd.), 100 nM miR-186-5p mimic (5′-CAAAGAAUUCUCCUUUUGGGCU-3′; Shanghai GenePharma Co., Ltd.), or CADM2 plasmid for 48 h, using Lipofectamine^®^ 2000 reagent (Invitrogen, USA), according to the manufacturer’s protocol. Subsequently, quantitative reverse transcription polymerase chain reaction (qRT-PCR) was performed to evaluate efficiency of cell transfection.

### 2.4 qRT-PCR

The RNA content was isolated from cells using TRIzol® reagent (Life Technologies, USA) according to the manufacturer’s protocol. Then, the total RNA was reverse transcribed to cDNA using the PrimeScript RT Reagent Kit (TaKaRa, China). All reactions were conducted using the Prism 7000 Real-Time PCR system and SYBR qPCR Master Mix (Thermo Fisher Scientific, Inc., USA) according to the manufacturer’s protocol. Primer sequences were obtained from SANGON Biotech Co., Ltd., China. The following thermal cycling conditions were applied for qRT-PCR: initial denaturation for 5 min at 95°C, followed by 40 cycles of 10 s at 95°C and one cycle of 30 s at 60°C. GAPDH for mRNA and U6 for miRNA were used as the internal controls. The relative expression levels of WT1-AS, miR-186-5p, and CADM2 were analyzed using the 2^−ΔΔCq^ method [44]. Primer sequences were synthesized by Sangon Biotech (Shanghai, China) and are listed in Table 1.

**Table 1:** Primer sequences for PCR

Gene name	Sequences: 5′–3′
WT1-AS	Forward, 5′-GCCTCTCTGTCCTCTTCTTTG-3′
	Reverse, 5′-GCTGTGAGTCCTGGTGCTTA-3′
miR-186-5p	Forward, 5′-TCAAAGAATTCTCCTTTTGGGCT-3′
	Reverse, 5′-CGCTTCACGAATTTGCGTGTCAT-3′
CADM2	Forward, 5′-TCTATTCCAACAAGTCAGAAAATAATG-3′
	Reverse, 5′-CGCTTAGACTTGATTTTGACGG-3′
GAPDH	Forward, 5′-ATCACTGCCACCCAGAAGAC-3′
	Reverse, 5′-TTTCTAGACGGCAGGTCAGG-3′
U6	Forward, 5′-CTCGCTTCGGCAGCACA-3′
	Reverse, 5′-AACGCTTCACGAATTTGCGT-3′

### 2.5 MTT assay [45]

Transfected cells (10^4^ cells/well) were seeded in a 96-well plate overnight. Subsequently, 10 μL 3-(4,5-dimethyl-2-thiazolyl)-2,5-diphenyl-2-*H*-tetrazolium bromide (MTT) solution was added to each well, followed by incubation for 4 h. The purple formazan crystals were dissolved in 100 μL DMSO (Sigma-Aldrich, USA), and cell proliferation was subsequently measured at optical density (OD)_570_ for 10 min, using a microplate reader (Jupiter G19060; Dorval, Canada).

### 2.6 Transwell assay [46]

Transwell assays were conducted using Matrigel-free chambers (pore size, 8 μm; Costar; Corning Inc., USA) and Matrigel chambers, to study cell migration and invasion, respectively. Cells (2 × 10^4^) were plated into the upper chambers (Thermo Fisher Scientific, Inc., USA) and maintained in serum-free DMEM medium after transfection for 48 h. DMEM containing 10% FBS was added into the lower chambers. After 24 h, the migratory or invasive cells in the lower chambers were treated with 4% paraformaldehyde (Sigma) and 0.1% crystal violet (Beyotime Institute of Biotechnology, China) for 20 min and counted under a light microscope (magnification: ×100; Olympus Corporation) in five randomly selected fields.

### 2.7 Flow cytometry (FCM) analysis [47]

FCM analysis was performed to detect cell apoptosis using the Annexin V-FITC/PI apoptosis detection kit (Beyotime Institute of Biotechnology, China). Transfected cells were collected following trypsinization and resuspended in Annexin V-FITC Binding Solution. The cell suspension (100 µL) was cultured with 5 µL annexin V-FITC and PI (BD Biosciences, USA) according to the manufacturer’s protocol. Stained cells were counted using a FACSCalibur flow cytometer (BD Biosciences, USA) and Kaluza Analysis (version 2.1.1.20653; Beckman Coulter, Inc., USA).

### 2.8 Western blotting [48]

Transfected cells were separated using radioimmunoprecipitation assay buffer (Beyotime Institute of Biotechnology, China) and subsequently centrifuged at 4°C for 15 min to collect the total protein content. Proteins were quantified using the BCA Protein Assay Kit (Beyotime Institute of Biotechnology, China) and loaded in 10% sodium dodecyl-sulfate polyacrylamide gel electrophoresis gel. The separated samples were transferred onto polyvinylidene fluoride membranes and incubated with 5% skim milk in PBS-Tween 20 (PBST) solution for 1 h. The membranes were incubated with primary antibodies against CADM2 (cat. no. ab64873; 1:2,000; Abcam, UK), and GAPDH (cat. no. 5174; 1:1,000; Abcam, UK) overnight at 4°C. Subsequently, the membranes were washed with PBST and incubated with secondary antibodies (cat no. ab7090; 1:2,000; Abcam, UK). Finally, protein bands were quantified using an enhanced chemiluminescence substrate (Cytiva, USA).

### 2.9 Statistical analysis

SPSS software (version 20.0; IBM Corp., USA) was used for statistical analyses. Data are presented as the mean ± SD from three independent experiments. We used the D method of the normality test (Kolmogorov–Smirnov test) to test the normality of the data in SPSS. Differences among multiple groups were estimated using one-way analysis of variance (ANOVA) and Student’s *t*-test. *P < 0.05 and **P < 0.01 indicated statistically significant differences.

## 3 Results

### 3.1 WT1-AS plasmid affects the viability, migration, and invasion of HTR-8/SVneo cells

**Figure 1:**
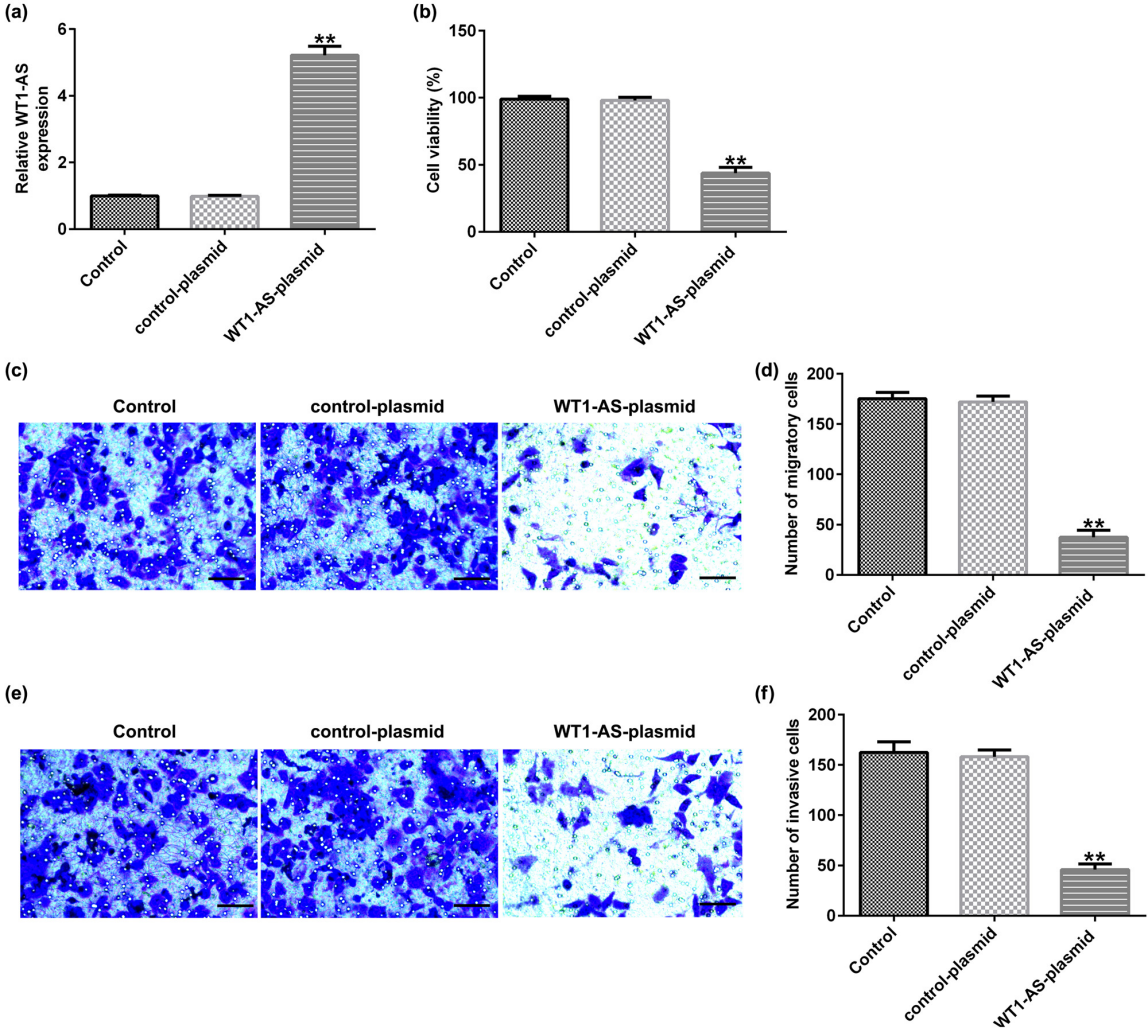
Effects of WT1-AS-plasmid on HTR-8/SVneo cell viability, migration, and invasion. (a) qRT-PCR analysis was used to determine the transfection efficiency of control plasmid and WT1-AS plasmid in HTR-8/SVneo cells. (b) The viability of HTR-8/SVneo cells was determined using the MTT assay. HTR-8/SVneo cell migration (c) and invasion (e) (magnification: ×100; bar = 100 μm) were assessed by Transwell assay. The number of migratory cells (d) and invasive cells (f) were determined. ^**^ *P* < 0.01 vs control plasmid. WT1-AS, WT1 antisense RNA.

To explore the role of WT1-AS in HTR-8/SVneo cells, control plasmid and WT1-AS plasmid were transfected into HTR-8/SVneo cells for 48 h. The results indicate that WT1-AS plasmid significantly increased WT1-AS expression (Figure 1a) and inhibited viability (Figure 1b), migration (Figure 1c and d), and invasion (Figure 1e and f) in HTR-8/SVneo cells, compared to that in the control group.

### 3.2 WT1-AS plasmid affects apoptosis of HTR-8/SVneo cells

**Figure 2:**
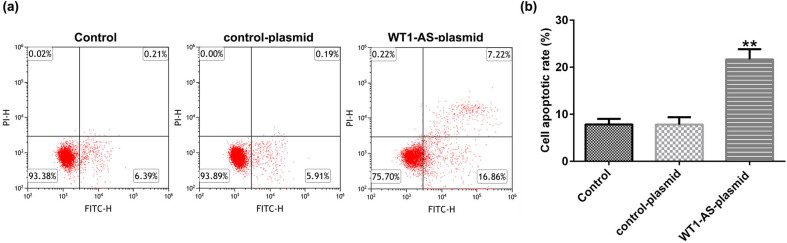
Effects of WT1-AS plasmid on apoptosis of HTR-8/SVneo cells. (a and b) Flow cytometry analysis of apoptotic HTR-8/SVneo cells. ^**^ *P* < 0.01 vs control plasmid. WT1-AS, WT1 antisense RNA.

FCM assay was performed to explore the effect of WT1-AS in HTR-8/SVneo cells apoptosis. FCM assay demonstrated that transfection with WT1-AS plasmid significantly promoted apoptosis of HTR-8/SVneo cells (Figure 2a and b).

### 3.3 MiR-186a-5p directly targets WT1-AS

**Figure 3:**
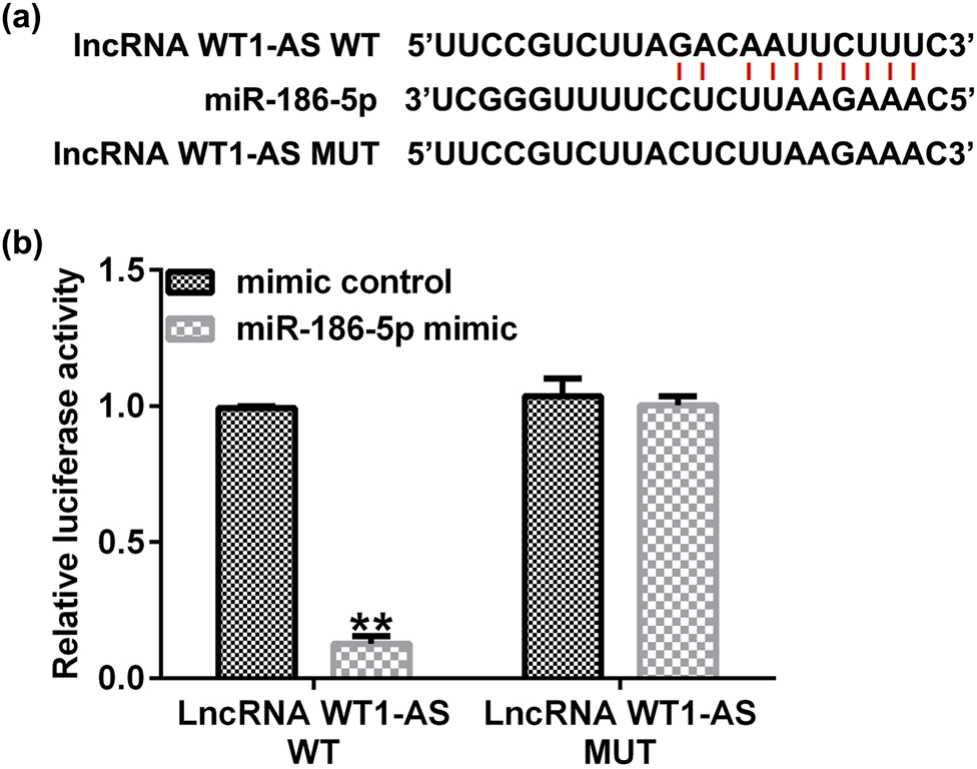
miR-186-5p directly targets lncRNA WT1-AS. (a) A schematic of miR-186a-5p binding site in lncRNA WT1-AS 3′-UTR. (b) The interaction between miR-186a-5p and lncRNA WT1-AS was confirmed via dual-luciferase reporter assay. ^**^ *P* < 0.01 vs mimic control. miR, microRNA; lncRNA, long noncoding RNA; WT1-AS, WT1 antisense RNA; WT, wild-type; MUT, mutant; UTR, untranslated region.

To analyze the mechanism of the role of WT1-AS in HTR-8/SVneo cells, the target relationship between WT1-AS and miR-186-5p was determined. StarBase analysis revealed a binding site between WT1-AS and miR-186-5p (Figure 3a), and this association was confirmed via the dual-luciferase reporter assay (Figure 3b). Compared with cells co-transfected with WT1-AS-WT and mimic control, the luciferase activity of cells co-transfected with WT1-AS-WT and miR-186-5p mimic significantly reduced (Figure 3b). While there were no significant differences in the luciferase activity of cells co-transfected with WT1-AS-WT and mimic control, and the cells co-transfected with WT1-AS-WT and miR-186-5p mimic were found.

### 3.4 WT1-AS negatively regulates miR-186a-5p expression in HTR-8/SVneo cells

**Figure 4:**
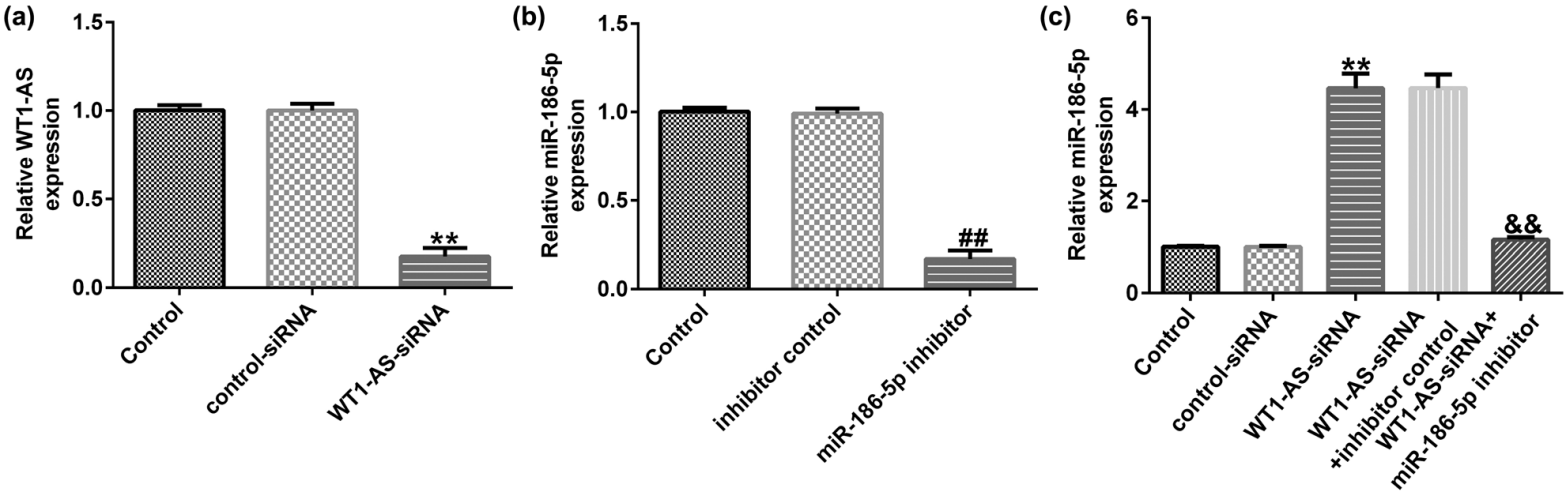
WT1-AS knockdown upregulates miR-186a-5p expression in HTR-8/SVneo cells. (a) qRT-PCR analysis of WT1-AS in HTR-8/SVneo cells transfected with control siRNA or WT1-AS siRNA. (b) Determination of miR-186-5p levels in HTR-8/SVneo cells transfected with inhibitor control or miR-186-5p inhibitor. (c) mRNA expression of miR-186-5p in HTR-8/SVneo cells transfected with control siRNA, WT1-AS-siRNA, WT1-AS-siRNA + inhibitor control, or WT1-AS-siRNA + miR-186-5p inhibitor was determined using qRT-PCR. ^**^ *P* < 0.01 vs control siRNA; ^##^ *P* < 0.01 vs inhibitor control; ^&&^ *P* < 0.01 vs WT1-AS-siRNA + inhibitor control. WT1-AS, WT1 antisense RNA; miR, microRNA; RT-qPCR, reverse transcription-quantitative PCR; si, small interfering.

We then explored whether WT1-AS could regulate miR-186a-5p expression in HTR-8/SVneo cells. qRT-PCR analysis indicated that transfection with WT1-AS-siRNA significantly decreased WT1-AS expression in HTR-8/SVneo cells, compared to that in the control siRNA group (Figure 4a). Furthermore, downregulation of miR-186-5p notably decreased miR-186-5p levels in HTR-8/SVneo cells (Figure 4b). Moreover, transfection with WT1-AS-siRNA markedly increased miR-186-5p levels in HTR-8/SVneo cells, which was reversed after co-transfection with miR-186-5p inhibitor (Figure 4c).

### 3.5 WT1-AS-siRNA affects the function of HTR-8/SVneo cells by regulating miR-186-5p

**Figure 5:**
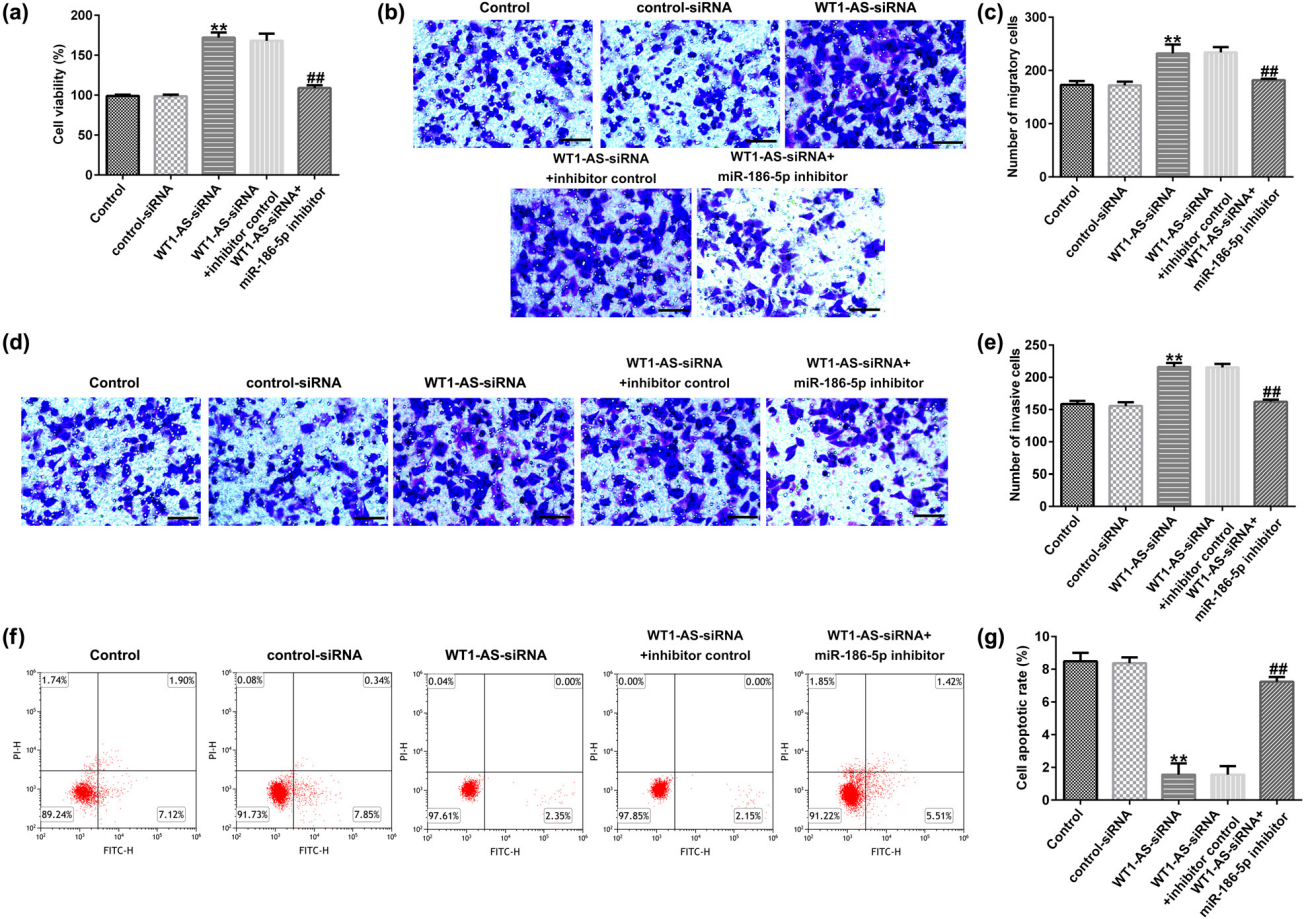
Effects of WT1-AS-siRNA on the function of HTR-8/SVneo cells. (a) Viability of HTR-8/SVneo cells was checked by the MTT assay. HTR-8/SVneo cell migration (b) and invasion (d) (magnification: ×100; bar = 100 μm) were evaluated using Transwell assay. The number of migratory cells (c) and invasive cells (e) were determined. (f and g) Flow cytometry was applied to assess apoptosis of HTR-8/SVneo cells. One-way ANOVA followed by Tukey’s post hoc test was used for data analysis. ^**^ *P* < 0.01 vs control-siRNA; ^##^ *P* < 0.01 vs WT1-AS-siRNA + inhibitor control. WT1-AS, WT1 antisense RNA; si, small interfering.

The results from MTT and transwell assays shown in Figure 5 suggest that WT1-AS-siRNA significantly promoted HTR-8/SVneo cell viability (Figure 5a), migration (Figure 5b and c), and invasion (Figure 5d and e), compared to that of control siRNA. Notably, transfection with WT1-AS-siRNA suppressed apoptosis in HTR-8/SVneo cells (Figure 5f and g), which was reversed after miR-186-5p inhibitor transfection.

### 3.6 CADM2 is a direct target of miR-186a-5p

**Figure 6:**
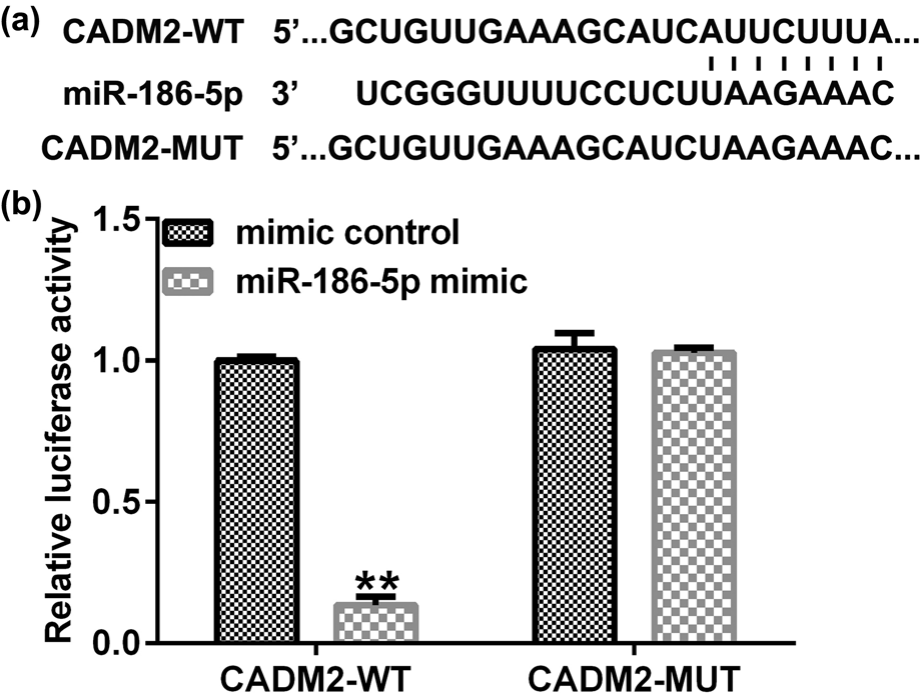
Correlation between miR-186-5p and CADM2. (a) StarBase analysis predicted the relationship between miR-186a-5p and CADM2. (b) The association between miR-186-5p and CADM2 was verified via the dual-luciferase reporter assay. ^**^ *P* < 0.01 vs mimic control. miR, microRNA; CADM2, cell adhesion molecule 2.

To confirm the underlying mechanism of miR-186a-5p in HTR-8/SVneo cells, the potential targets of imiR-186a-5p were investigated using TargetScan. TargetScan analysis revealed a binding site between CADM2 and miR-186a-5p (Figure 6a), and this association was confirmed via the dual-luciferase reporter assay (Figure 6b).

### 3.7 MiR-186a-5p negatively regulates CADM2 expression in HTR-8/SVneo cells

**Figure 7:**
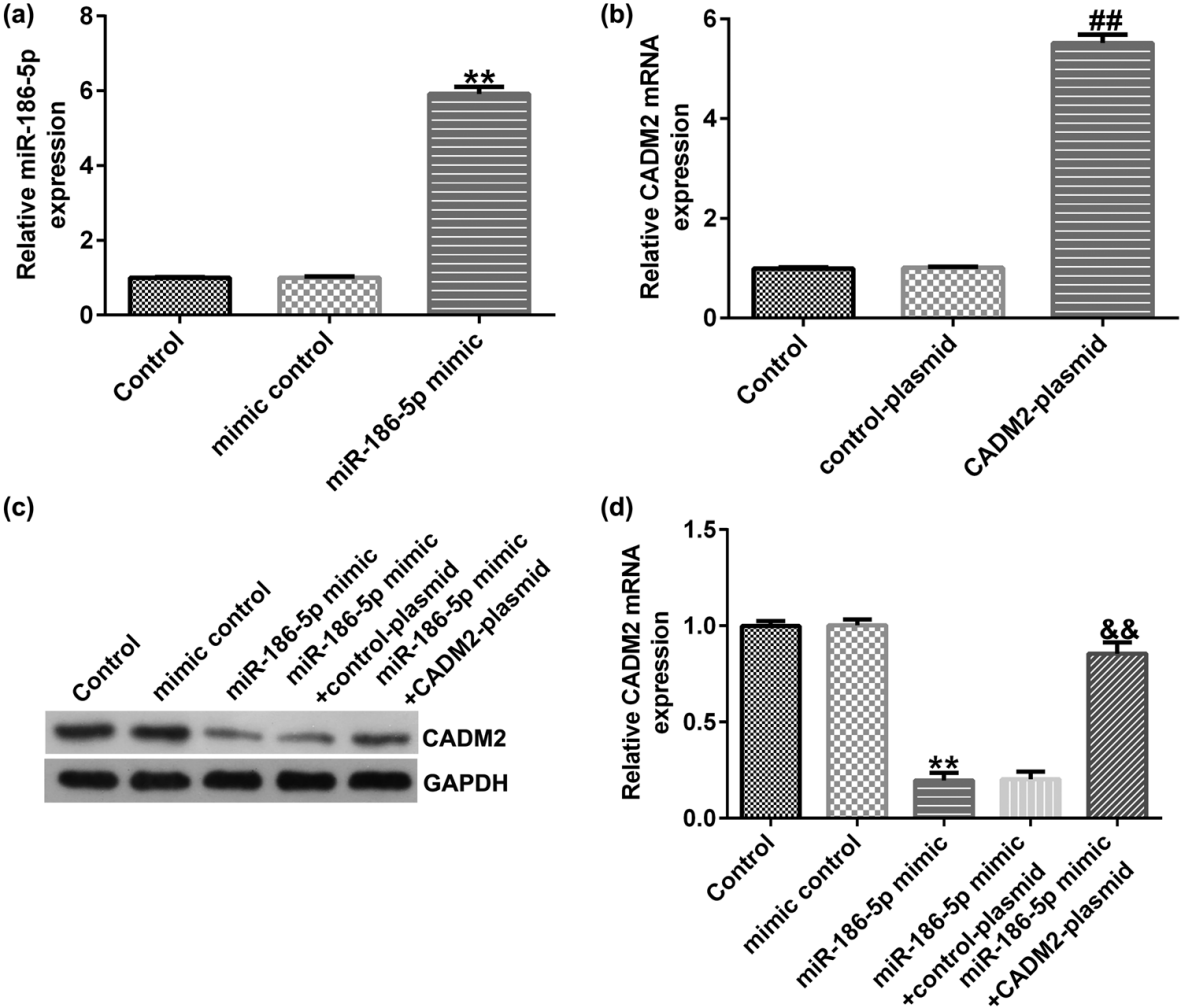
miR-186-5p negatively regulates CADM2 expression in HTR-8/SVneo cells. (a) qRT-PCR analysis of miR-186-5p in HTR-8/SVneo cells transfected with mimic control or miR-186-5p mimic. (b) qRT-PCR analysis was performed to detect CADM2 expression in HTR-8/SVneo cells transfected by control plasmid or CADM2 plasmid. (c and d) Determination of CADM2 expression levels in HTR-8/SVneo cells transfected by mimic control, miR-186-5p mimic, miR-186-5p mimic + control plasmid, or miR-186-5p mimic + CADM2 plasmid using western blot and qRT-PCR analyses. ^**^ *P* < 0.01 vs mimic control; ^##^ *P* < 0.01 vs control-plasmid; ^&&^ *P* < 0.01 vs miR-186-5p mimic + control-plasmid. miR, microRNA; CADM2, cell adhesion molecule 2; RT-qPCR, reverse transcription-quantitative PCR.

To confirm the regulatory effect of miR-186a-5p on HTR-8/SVneo cells, mimic control, miR-186a-5p mimic, control plasmid, CADM2 plasmid, miR-186a-5p mimic + control plasmid, and miR-186a-5p mimic + CADM2 plasmid were transfected into HTR-8/SVneo cells. qRT-PCR results indicated that transfection with miR-186-5p mimic and CADM2 plasmid significantly increased miR-186-5p and CADM2 levels in HTR-8/SVneo cells, respectively (Figure 7a and b). Notably, miR-186-5p mimic decreased CADM2 protein and mRNA expression levels in HTR-8/SVneo cells, which was reversed after co-transfection with CADM2 plasmid (Figure 7c and d).

### 3.8 MiR-186-5p mimic affects the function of HTR-8/SVneo cells by regulating CADM2

**Figure 8:**
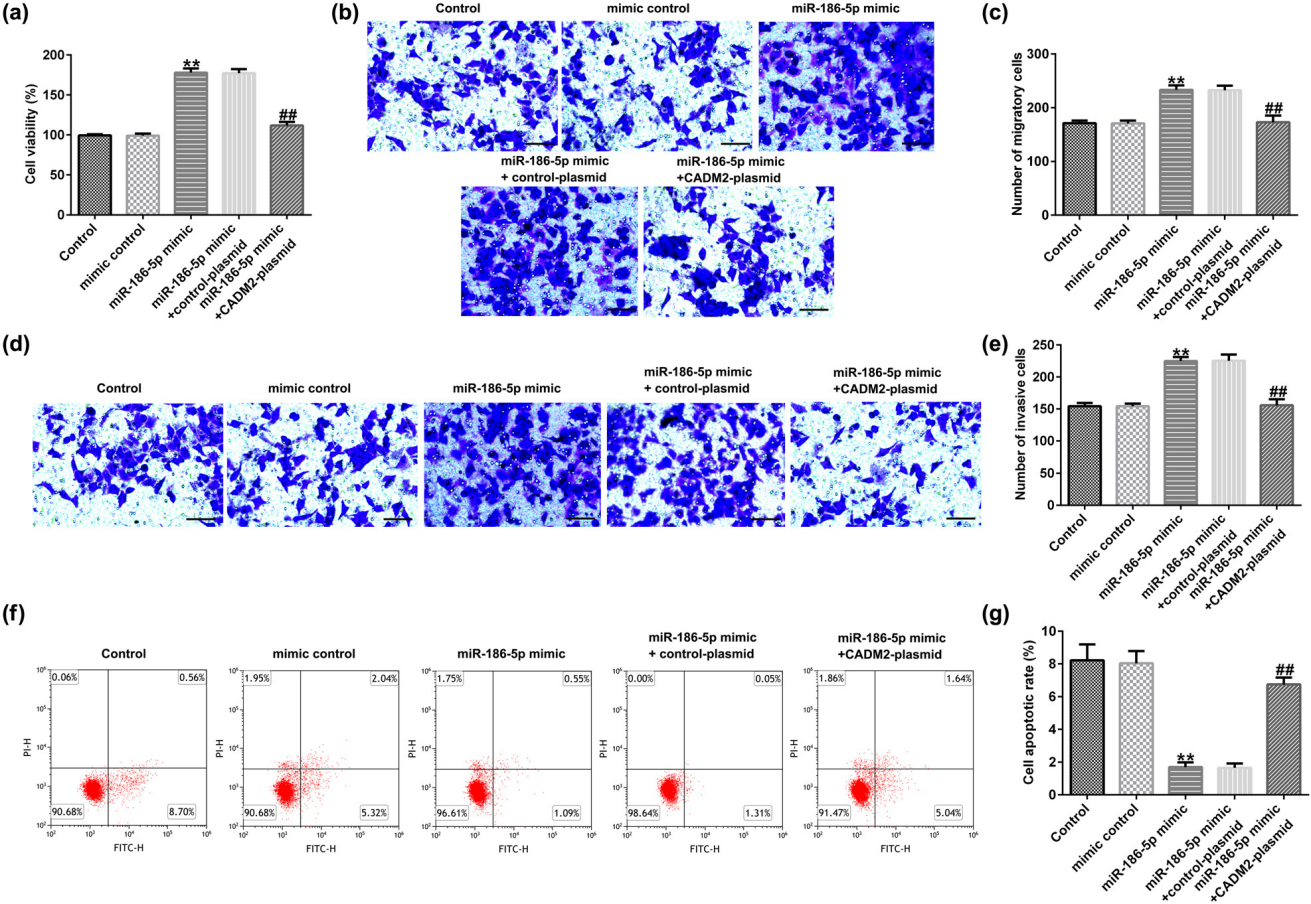
Effects of miR-186-5p mimic on the functions of HTR-8/SVneo cells. (a) Viability of HTR-8/SVneo cells was assessed using MTT assay. Transwell assay was used to analyze HTR-8/SVneo cell migration (b) and invasion (d) (magnification: ×100; bar = 100 μm). The number of migratory cells (c) and invasive cells (e) was determined. (f) Flow cytometry analysis of apoptosis in HTR-8/SVneo cells. (g) Quantification of apoptotic cells. ^**^ *P* < 0.01 vs mimic control; ^##^ *P* < 0.01 vs miR-186-5p mimic + control-plasmid. miR, microRNA.

Finally, we studied whether miR-186-5p mimic affects the function of HTR-8/SVneo cells by targeting CADM2. Transfection with the miR-186-5p mimic dramatically increased HTR-8/SVneo cell viability (Figure 8a) and enhanced the migratory (Figure 8b and c) and invasive (Figure 8d and e) abilities of cells, compared to that in the mimic control group. The FCM analysis indicated a notable decrease in apoptosis following transfection with the miR-186-5p mimic, compared to that of the mimic control group (Figure 8f and g).

### 3.9 WT1-AS-siRNA-reduced CADM2 expression in HTR-8/SVneo cells

**Figure 9:**
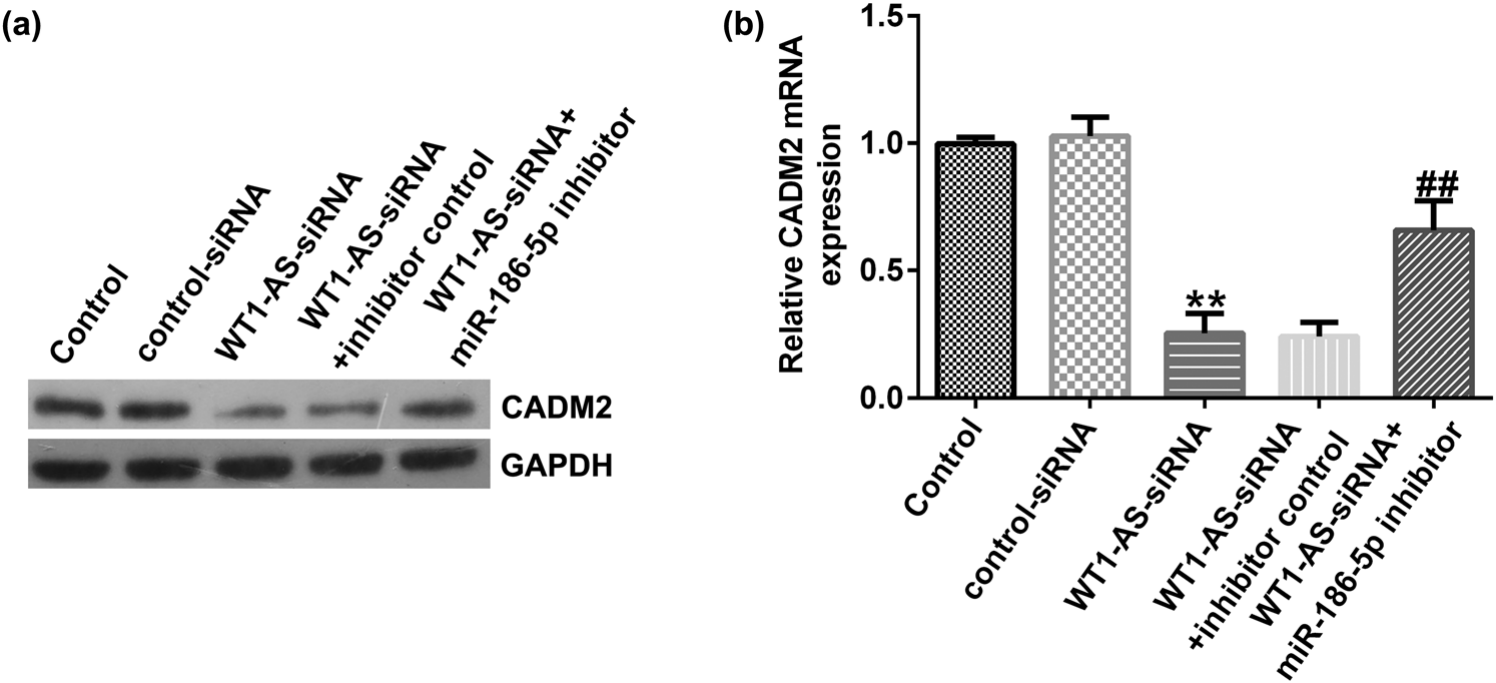
WT1-AS-siRNA-reduced CADM2 expression in HTR-8/SVneo cells. HTR-8/SVneo cells were transfected with control siRNA, WT1-AS siRNA, WT1-AS siRNA + inhibitor control, or WT1-AS siRNA + miR-186-5p inhibitor. (a) Protein expression of CADM2 in HTR-8/SVneo cells was determined using western blotting. (b) mRNA expression of CADM2 in HTR-8/SVneo cells was determined using RT-qPCR. ^**^ *P* < 0.01 vs control siRNA; ^##^ *P* < 0.01 vs WT1-AS siRNA + inhibitor control. WT1-AS, WT1 antisense RNA; si, small interfering; miR, microRNA; RT-qPCR, reverse transcription-quantitative PCR.

Finally, to explore the effect of WT1-AS-siRNA on CADM2 expression in HTR-8/SVneo cells, HTR-8/SVneo cells were transfected with control-siRNA, WT1-AS-siRNA, WT1-AS siRNA + inhibitor control, or WT1-AS siRNA + miR-186-5p inhibitor. The results indicated that compared with the control siRNA group, WT1-AS siRNA significantly reduced CADM2 protein and mRNA expression in HTR-8/SVneo cells, and this reduction was reversed by miR-186-5p inhibitor (Figure 9a and b).

## 4 Discussion

PE is a hypertensive condition affecting pregnant women. Its clinical manifestations vary; however, hypertension and proteinuria are common symptoms [49]. PE and placental insufficiency are closely related. The placenta is essential for the fetal development as fetal blood and maternal blood exchange nutrients through it. The placenta has two main functions, endocrine signaling and invasion, and these functions depend on the EVTs [50]. The main reason behind the onset of PE is the failure of remodeling of the uterine spiral artery caused by insufficient EVT infiltration.

HTR-8/SVneo cells, developed by Graham et al. [41], have been widely used to study PE *in vitro* [51–53]. Thus, this study used HTR-8/SVneo cells to study PE *in vitro.* In recent years, more and more studies have shown that lncRNA plays a key role in PE [20,25–27]. lncRNA NEAT1 silencing improves Treg/Th17 imbalance in PE via the miR-485-5p/AIM2 axis [21]. lncRNA MEG3 has been reported to inhibit trophoblast invasion [26]. It has been confirmed that lncRNA CRNDE could regulate trophoblast cell proliferation, invasion, and migration through modulating miR-1277 [26]. In this study, we used HTR-8/SVneo cells to explore the role of WT1-AS in the function of placental EVTs. First, through database analysis, we found that WT1-AS is expressed in the placenta (https://www.ncbi.nlm.nih.gov/gene/51352). In addition, we first confirmed that lncRNA WT1-AS, miR-186-5p, and CADM2 were stably expressed in HTR-8/SVneo cells (Figures 4 and 7) and then proceeded with subsequent experiments. We observed that lncRNA WT1-AS inhibited various functions of HTR-8/SVneo cells, including cell growth, migration, and invasion, and promoted apoptosis.

Accumulating evidence suggests that lncRNAs can act as miRNA sponges, repressing miRNA expression and regulating mRNA expression at the posttranscriptional level [24,54]. To further determine the molecular mechanism of WT1-AS in placental EVTs, the binding sites between WT1-AS and miR-186-5p were predicted and verified. The results indicated a negative correlation between miR-186-5p and WT1-AS levels in HTR-8/SVneo cells. MiR-186-5p has also been shown to be involved in PE [37,38]. Gusar et al. revealed that miR-186-5p is upregulated in blood plasma during early onset PE [37]. Hsa_circ_0001326 suppressed human trophoblast SWAN71 viability by regulating the miR-186-5p/p27 kip1 axis [38]. In this study, WT1-AS knockdown promoted the function of placental EVTs by upregulating miR-186-5p expression. The present study also verified the relationship between miR-186-5p and CADM2. CADM2, a protein-coding gene, acts as a tumor suppressor in cancer through inhibiting cell migration and invasion [39,40]. He et al. reported that CADM2 could inhibit human renal clear cell carcinoma by promoting DNA promoter methylation and/or loss of heterozygosity [55]. To determine whether miR-186-5p regulates the proliferation, migration, and invasion of placental EVTs by regulating CADM2, miR-186-5p and CADM2 were upregulated in HTR-8/SVneo cells. The results indicated that CADM2 negatively regulates miR-186-5p expression. In addition, miR-186-5p promoted the function of HTR-8/SVneo cells after the downregulation of CADM2 expression. In this study, the mechanism by which CADM2 affected the function of HTR-8/SVneo cells still needs to be further explored.

The current study had some limitations. For example, EVTs fail to invade and remodel the spiral arteries in the first trimester of pregnancy [14], and HTR-8/SVneo cell is a model of first-trimester EVT [41]. Thus, our study models process that take place in the first trimester (EVT-mediated remodeling of the spiral arteries), but PE is not evident until the mid-second trimester. But in fact, miRNA biomarkers for PE can be detected in maternal circulation as early as the first trimester, although PE is not evident until later in pregnancy [56–58]. Besides, this work was performed using HTR-8/SVneo cells and needs validation using primary human first trimester EVTs. Whether the change of WT1-AS/miR-186a-5p/CADM2 axis affect cell cycle in trophoblasts was not investigated. We also did not perform migration and invasion assays on HTR-8/SVneo cells with cell cycle arrest to explain the effects of proliferation. Altered angiogenic factors are known contributors to the pathogenesis of PE, and this study did not analyze the effect of WT1-AS on angiogenic factor receptors. In addition, there is a significant difference between the conditions of the *in vitro* experiments and those of PE. Moreover, the role of lncRNA WT1-AS/miR-186-5p/CADM2 axis in the function of other placental EVTs (such as primary human first trimester EVTs and other trophoblast cell-lines such as JEG-3, BeWo cells or JAR cells) needs to be explored, as does the role of lncRNA WT1-AS/miR-186-5p/CADM2 axis in PE animal models. The expression of lncRNA WT1-AS in PE and non-PE patients should be investigated. Moreover, roles of lncRNA WT1-AS/miR-186-5p/CADM2 in PE patients, and the relationship between their expression and the clinicopathological parameters of PE patients, also need to be determined. We will address these issues in the future.

## 5 Conclusion

Our findings suggest that lncRNA WT1-AS regulates HTR-8/SVneo cell proliferation and invasion through the miR-186-5p/CADM2 axis, and participates in PE, indicating that targeting the miR-186-5p/CADM2 axis can provide novel opportunities for patients with PE.
